# Marital duration in China: Trends and gender differences, 1982–2015

**DOI:** 10.3389/fpubh.2022.831147

**Published:** 2022-09-14

**Authors:** Yingrun Zhu, Xiao Yu, Quanbao Jiang

**Affiliations:** ^1^School of Finance and Economics, Guangdong Polytechnic Normal University, Guangzhou, China; ^2^Northeast Asia Research Center, Jilin University, Changchun, China; ^3^School of Public Policy and Administration, Institute for Population and Development Studies, Xi'an Jiaotong University, Xi'an, China

**Keywords:** marital duration, trends, gender differences, Sullivan's method, life expectancy

## Abstract

**Objectives:**

With the postponement in age at marriage, increase in life expectancy, and acceptance of divorce in China, the marital duration in each state has changed gradually. This study seeks to depict the trends and gender differences of marital duration in China from 1982 to 2015.

**Design:**

We calculated and depicted the trends of and gender differences in marital duration, including single, marriage, divorce, widowhood duration and the proportion of each duration to the remaining life expectancy at 15.

**Results:**

The single duration of Chinese men and women was slightly reduced and then extended, with that of men longer than women, showing a narrowing trend in gender difference. The marriage duration of Chinese men and women is lengthening, with that of women longer than men, demonstrating a widening gap in gender difference. However, the proportion of marriage duration in life expectancy at age 15 increased and then declined, with that of men higher than women. The divorce duration of Chinese men and women slightly reduced and then lengthened, but men tend to stay divorced for longer periods than women, and the gender difference is narrowing. The widowhood duration of Chinese men and women is shortening, with women having longer widowhood than men, and the gender difference has been shrinking.

**Conclusions:**

With the socio-economic and demographic transition, the marital duration in each state has changed gradually, and will have an important impact on fertility level and pension burden.

## Introduction

China's traditional agricultural society was characterized by the four phenomenon in marriage, namely, early marriage, universal marriage, low divorce rate and high widower proportion for older adults. However, with the rapid development of industrialization, the modernization process of Chinese society has been advancing rapidly. Gender roles, gender ideology, inter-family relationships and health levels have changed dramatically. The first is the equalization of gender roles. In the traditional agricultural society, there was a large gap between men and women in educational attainment and income level, and marriage was the main way for women to obtain economic support (1). With the acceleration of China's modernization process, especially after the expansion of college enrollment beginning in the late 1990s, women's education level and labor participation rate have improved rapidly, and the gap between men and women in social status, income and career promotion has been narrowing, and the value of marriage for professional women has been decreasing (2). The second is the radical change in gender ideology. In traditional agricultural society, premarital and extramarital sex were strongly condemned by morality, and divorce was extremely rare (3). However, in modern society, premarital cohabitation and divorce are gradually being accepted (1, 4). The third is the change in inter-family relationship. In traditional agricultural society, the core of family was the parent-child relationship (5). After entering modern society, the marital relationship has become the core of the family (6). The fourth is the health improvement. The life expectancy of Chinese population increased rapidly from 67.77 years in 1982 to 76.34 years in 2015 (7). The proportion of widowers dropped, from 7.12% in 1982 to 5.51% in 2015 among people aged 15 and above (8, 9).

In the transition process from traditional agricultural society to modernization, marriage status has changed greatly in both developed and developing countries (1, 10–13). Since 1980, the marital status of Chinese people has undergone tremendous change with the following four characteristics.

Firstly, the delay in marriage leads to a longer single duration and higher proportion of never-married young people and longer single duration. The rising cost of marriage, higher education levels and the urbanization of the population are the main factors behind the delay in first marriage. Marketization and economic development have improved the economic foundation for marriage, and many people need to accumulate more economic resources to get married (14). Enrollment expansion of higher education prolongs the schooling life and delays the timing for young people to enter the marriage market (15). The average age of first marriage of the population with higher education is higher than that of the population without higher education (16). Urban population receives longer years of education, faces higher housing prices and more employment opportunities than rural population, so the average age of first marriage of urban population is higher than that of rural population (17). The mean age at first marriage for men and women gradually changed from 23.57 and 22.02 years old in 1990 to 25.63 and 23.89 years old in 2021, respectively (18). The expected number of years that people aged 15 remain never-married increased between 1990 and 2010, with that of men exceeding that of women (19). The current proportion of never-married women is small (20), but will increase in the future (21, 22). The proportion of never-married men will also increase (23). The expected number of years to remain never-married will continue to increase for Chinese people.

Secondly, although the proportion of married young people has declined, increased life expectancy directly lengthens marriage duration. Higher education has a great impact on the marriage rate, and the marriage rate of undergraduates and postgraduates has declined significantly (24). However, as life expectancy increases, the proportion of middle-aged and older people, especially women, who are married has increased. The marriage duration of men and women grew from 38.59 and 39.43 years in 1982 to 41.49 and 43.18 years in 1995, respectively, while the gap in gender difference widened (25).

Thirdly, the rising divorce rate has led to an increased divorced population and an extension of the expected number of years of people in divorce. With the modernization process, China's social and economic development has made great progress. Women's education level and social and economic status have been continuously improved. The ideology of marriage and family has undergone great changes, and the divorce rate has been rising (26–28). The crude divorce rate in China rose from 0.18‰ in 1978 to 3.36‰ in 2019 (28). The proportion of divorce among men and women aged 15 and older increased from 9.17 and 2.55‰ in 1982 to 19.36 and 14.93‰ in 2015, respectively (8, 9). As life expectancy lengthens and the proportion of divorce increases, the number of years in divorced status also increases.

Fourthly, increased life expectancy and a smaller proportion of widowhood result in fewer years of people in widowhood. The life expectancy of Chinese men and women grew from 66.28 and 69.27 years in 1982 to 73.64 and 79.43 years in 2015, respectively (8, 9). In China, the proportion of widowers and widows aged 60 and above decreased from 26.90 and 58.09% in 1982 to 14.05 and 32.07% in 2015, respectively (8, 9). The expected number of years of widowhood for men and women in 2010 was about 11 and 15 years, respectively (29). Since the expected number of years of widowhood in the U.S. decreases as life expectancy increases (30), it can be predicted that the expected years of widowhood in China are also decreasing.

Several studies have analyzed the change of expectation years of different marital status in China (19, 29), but the exact expected number of years of men and women in never-married, currently-married, divorced, and widowed statuses since the 1980s have not been systematically studied and clarified in light of trend changes and gender differences. Using National Population Census and National one Percent Population Sample Survey in China since the 1980s, this paper, firstly, analyzes the trends in the expected number of years of Chinese men and women in never-married, currently married, divorced, and widowed statuses and developing trends of the proportion of each expected number of years in life expectancy. Secondly, gender differences in the expected number of years of Chinese people in never-married, married, divorced, and widowed statuses are analyzed, respectively. Thirdly, the decomposition method is used to analyze the causes of changed duration of the four marital statuses and gender differences. By studying the above issues, we hope to have an overall understanding of the trends of the expected number of years of Chinese people in the four marital statuses and gender differences to provide a reference for understanding marital problems, fertility levels, and family breakdown risks in China.

## Methods

### Marital duration measurement

In this study, the average number of years of never-married, currently married, divorced, and widowed statuses are collectively referred to as marital duration. Marital duration is an index measure of the expected number of years people stay in different marital statuses. Scholars have used life tables to measure this for people in married and widowed statuses (29–31). Wei et al. constructed a net nuptiality table to measure the average duration of never-married people with never-married status (19). Zeng et al. based on multi-source data, constructed a multi-decrement life table to calculate the expected number of years in 1970–2002 of American men and women above 15 years of age in seven marital statuses, namely, never-married and not cohabited, never-married but cohabiting, married, divorced and not cohabited, divorced but cohabiting, widowed and not cohabited, widowed but cohabiting (32). The transition probabilities among the seven statuses were calculated and the gender differences, racial differences, and trends were analyzed.

The Sullivan Method (33) can be used to measure the expected number of years of people in multiple statuses and has been applied to studies on measuring healthy life expectancies (34–36), happiness life expectancies (37, 38), floating Life Expectancy (39), working Life Expectancy (40), empty-nest Life Expectancy (41), and marriage duration (25, 42). Referring to the Sullivan Method, this study combined mortality rates with the proportion of single, marriage, divorce, and widowhood, respectively, and constructed the single, marriage, divorce, and widowhood duration to measure the expected number of years of people in single, married, divorced and widowed statuses.

(1) Single duration: average number of years of never-married during whole life span per person.
(2) Marriage duration: average number of years of currently married during whole life span per person.
(3) Divorce duration: average number of years of divorced during whole life span per person.
(4) Widowhood duration: average number of years of widowed during whole life span per person.

Taking marriage duration as an example, if we denote as the expected number of years at the age of *x* and *m*_*x*_ as the marriage duration at the age of *x*, then according to the life table function, the formula of expected number of years at the age of *x* is $e_{x}=(\sum_{y=x}^{\omega -n}{}_{n}L_{y}+L_{\omega +})/l_{x}$. By replacing the person-year of survival _*n*_*L*_*y*_ in the formula of expected number of years with the married person-year of survival _*n*_*L*_*y*_(*M*), the marriage duration $m_{x}=\left[\sum_{y=x}^{\omega -n}{}_{n}L_{y}(M)+L_{\omega +}(M)\right]/l_{x}$. According to the Sullivan Method, we assume _*n*_*L*_*y*_(*M*) =_*n*_π_*y*_·_*n*_*L*_*y*_, where _*n*_π_*y*_ means the proportion of married people accounting for the total population at the age of *y* to *y*+*n*. Therefore, the formula for marriage duration at the age of *x* can be expressed as $m_{x}=\left[{\sum_{y=x}^{\omega -n}{}_{n}\pi_{y}}_{n}L_{y}+\pi_{\omega +}L_{\omega +}\right]/l_{x}$. Similarly, single, divorced, and widowhood duration can be defined.

### Decomposition of period and gender differences in marital duration

Andreev et al. aimed to study life expectancy defined by the Sullivan method (34). They constructed a decomposition model of life expectancy differences to study the effects of mortality differences and health ratio differences at different ages on differences in healthy life expectancy. This method has also been used to decompose the change in healthy life expectancy in Japan from 1986 to 2004 (35) and the change in happiness life expectancy in the United States from 1970 to 2000 (37). In this paper, we referred to the method of Andreev et al. (34) and constructed a two-factor decomposition model of period differences (or gender differences) in marital duration. With that, decompose the contributions of period differences (or gender differences) in mortality, the proportion of marital status in life expectancy, and the period differences (or gender differences) in marital duration by age. Taking gender difference in marriage duration as an example, the specific formula is as follows.

Assuming that men and women are represented as *j* = 1, 2, respectively, the gender difference in marriage duration of people at the initial age can be decomposed as:


(1) $$m_{x_{0}}^{2}-m_{x_{0}}^{1}=\sum_{x=x_{0}}^{\omega +}{}_{n}\lambda_{x}+\sum_{x=x_{0}}^{\omega +}{}_{n}a_{x},$$

Where the contribution of gender differences in death levels from age *x* to age *x*+*n* is


(2) $$\begin{array}{l} {}_{n}\lambda_{x}=\frac{1}{4}\left({}_{n}l_{x}^{1}{+}_{n}l_{x}^{2}\right)\left({}_{n}\pi_{x}^{1}{+}_{n}\pi_{x}^{2}\right)\left({}_{n}P_{x}^{\text{2}}{-}_{n}P_{x}^{\text{1}}\right)\\ \;+\frac{n}{2}\left({}_{n}m_{x+1}^{1}\cdot_{n}l_{x}^{2}{+}_{n}m_{x+1}^{2}\cdot_{n}l_{x}^{1}\right)\left({}_{n}q_{x}^{1}{-}_{n}q_{x}^{2}\right); \end{array}$$

The contribution of gender differences in marriage proportion from age *x* to age *x*+*n* is


(3) $${}_{n}\alpha_{x}=\frac{1}{4}\left({}_{n}l_{x}^{1}{+}_{n}l_{x}^{2}\right)\left({}_{n}P_{x}^{1}{+}_{n}P_{x}^{2}\right)\left({}_{n}\pi_{x}^{1}{-}_{n}\pi_{x}^{2}\right).$$

### Data

Data used in this study include death data and marital data. China conducted National Censuses in 1982, 1990, 2000, and 2010 (8, 18, 43, 44) and National One-percent Sample Census in 1987, 1995, 2005, and 2015 (9, 45–47). The public aggregated data provided age-gender-marital status-specific data.

Quality issues are apparent in the death data of both the Chinese National Census and the National One-percent Sample Census. Chinese National Census data in 1982 (48), 1990 (49), 2000 (49–51), and 2010 (52, 53) had underreporting and misreporting, especially for children and the elderly which had no significant effect on this study. The quality of the elderlies' mortality data in 1982 was good, except in the Xinjiang Uygur Autonomous Region, where mortality data for older men were seriously misreported (54). The 1990 mortality data for the elderly were reliable. The 2000 and 2010 death data for the elderly had relatively good quality except for provinces with large minority populations (Xinjiang and Tibet autonomous regions, Yunnan, and Guizhou), where the quality of mortality data of those aged 90 and older were poor (53). Through studies with the DCMD model life table, Li et al. found that the underreporting of deaths among those aged 60–89 years were 2.3 and 7.0% for men and women, respectively, in 2010 (55).

Life expectancy, as computed directly from census data, is higher due to serious underreporting of mortality data, especially in infant and child deaths. The National Bureau of Statistics adjusted and published sex-specific life expectancies for census and sample survey years, which is lower than life expectancy calculated directly from census data, but the National Bureau of Statistics did not provide the corresponding adjustment and calculation method. In view of this, we used the sex-specific life expectancy published by National Bureau of Statistics as in presented in Table 1. In this paper, we adopted the general model of the United Nations model life tables and interpolated the life expectancy. With the interpolated life expectancy we obtained the general life table and corresponding age-specific death rates and probabilities. This process was fulfilled with the software PADIS-INT developed by the China Population and Development Research Center.

**Table 1:** Life expectancy in China, 1982–2015.

**Year**	**Men**	**Women**
1982	66.28	69.27
1987	66.68	70.13
1990	66.84	70.47
1995	68.24	71.90
2000	69.63	73.33
2005	70.83	75.25
2010	72.38	77.37
2015	73.64	79.43

Marriage data include 1-year age groups data by sex and marital status from the 1982, 1990, 2000, and 2010 National Census data and 1987, 1995, 2005, and 2015 National One-percent Sample Census. For population censuses and inter-census 1 percent population surveys from 1982 to 2015, data were aggregated on the status of marriages by sex and age between the ages of 15 and 59. However, in some years data between the ages of 60 or 65 and over were grouped together. Therefore, we calculated the proportions of being single, married, divorced and widowed for 15–59 years old age group by sex and 5-year age group in each year. The proportions of being single, married, divorced and widowed by sex for people aged 60 and above were for the total age range and not disentangled by age group. Although this treatment may cause some errors in the results, it does not affect the trend judgment. We took 15 as the starting age and excluded the marriage under 15.

## Results

Based on the above formula and data, the trends and gender differences in marital duration for men and women during 1982–2015 are discussed below.

### Trends and gender differences in single duration and proportion

**Figure 1:**
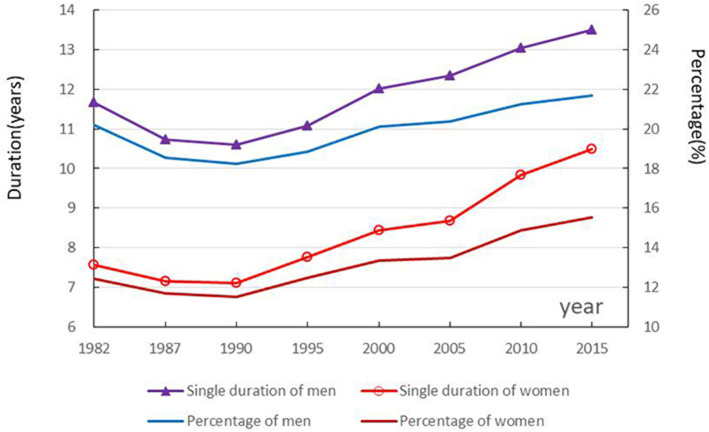
Trends in single duration and proportion among 15-year-olds.

The single duration and its proportion in life expectancy for men and women at age 15 in China declined and then increased from 1982 to 2015, with the period of men longer than women and the proportion of men larger than women. As shown in Figure 1, the single duration for 15-year-old men and women declined from 11.67 and 7.56 years in 1982 to 10.59 and 7.11 years in 1990, increasing to 13.48 and 10.49 years in 2015, respectively. The single duration proportion in life expectancy declined from 20.20 and 12.45% in 1982 to 18.24 and 11.55% in 1990, increasing to 21.67 and 15.56% in 2015, respectively. The gender difference gradually declined from 7.75% in 1982 to 6.11% in 2015.

During 1982–1990, single duration decreased by 1.08 and 0.45 years, respectively, among 15-year-old men and women in China, but during 1990–2015, the period increased by 2.89 and 3.38 years, respectively. The decomposed results provided in Table 2 show that changes in the never-married proportion have a large positive effect on those in single duration. Decreases in the proportion of never-married men and women contributed to the reduction of 1.09 and 0.46 years of single duration from 1982–1990. Increases in the proportion of never-married men and women supported the increase of 2.86 and 3.41 years of single duration. Declining mortality had a smaller positive effect on longer single duration.

**Table 2:** Decomposition of marital duration period differences among 15-year-olds.

**Gender**	**Initial year**	**Initial duration**	**Difference**	**Effect of period differences in mortality**	**Effect of period differences in the proportion 15–100**+**years-old**			**Ending duration**	**Ending year**
**Single**									
Men	1982	11.67	−1.07	0.01	−1.09			10.59	1990
Women	1982	7.56	−0.45	0.01	−0.46			7.11	1990
Men	1990	10.59	3.02	0.15	2.86			13.61	2015
Women	1990	7.11	3.44	0.03	3.41			10.55	2015
Men	1982	11.67	1.81	0.17	1.64			13.48	2015
Women	1982	7.56	2.93	0.04	2.89			10.49	2015
**Marriage**					15–44	45–100+	15–100+		
Men	1982	40.28	4.46	3.49	−1.86	2.83	0.96	44.74	2015
Women	1982	40.89	6.94	3.95	−3.13	6.11	2.99	47.83	2015
**Divorce**									
Men	1982	0.69	−0.14	0.04	−0.18			0.56	1987
Women	1982	0.19	−0.01	0.00	−0.01			0.18	1987
Men	1987	0.56	0.55	0.06	0.49			1.11	2015
Women	1987	0.18	0.72	0.04	0.68			0.90	2015
Men	1982	0.69	0.42	0.10	0.31			1.11	2015
Women	1982	0.19	0.71	0.04	0.67			0.90	2015
**Widowhood**									
Men	1982	5.12	−2.22	0.74	−2.96			2.90	2015
Women	1982	12.11	−3.91	2.65	−6.56			8.20	2015

The gender difference in single duration among 15-year-old people in China gradually decreased from 4.11 years in 1982 to 2.99 years in 2015. The decomposed results provided in Table 3 shows that the gender difference in the proportion of never-married people has a larger positive effect on gender difference in the single duration, decreasing from 4.16 years in 1982 to 3.08 years in 2015; the gender difference in mortality has a smaller negative effect on gender difference in the single duration, increasing from 0.05 years in 1982 to 0.09 years in 2015.

**Table 3:** Decomposition of marital duration gender differences among 15-year-olds.

	**1982**	**1987**	**1990**	**1995**	**2000**	**2005**	**2010**	**2015**
**Single**								
Effect of gender differences in mortality	0.05	0.05	0.06	0.06	0.07	0.08	0.09	0.09
Effect of gender differences in proportion of the never-married	−4.16	−3.63	−3.54	−3.38	−3.63	−3.72	−3.30	−3.08
Gender differences in single duration	−4.11	−3.58	−3.48	−3.32	−3.57	−3.64	−3.21	−2.99
**Marriage**								
Effect of gender differences in mortality	1.80	1.98	2.23	2.35	2.46	2.90	3.30	3.94
**Impact of gender differences in the married proportion**								
15–49 years old	3.64	3.06	2.88	2.65	2.74	2.83	2.38	2.33
50–100+ years old	−4.83	−4.60	−4.23	−3.85	−3.48	−3.30	−3.43	−3.18
15–100+ years old	−1.19	−1.54	−1.35	−1.19	−0.74	−0.47	−1.05	−0.84
Gender differences in marriage duration	0.61	0.44	0.87	1.15	1.72	2.43	2.25	3.10
**Divorce**								
Effect of gender differences in mortality	0.03	0.03	0.03	0.03	0.02	0.03	0.04	0.06
Effect of gender differences in divorce proportion	−0.54	−0.40	−0.41	−0.34	−0.30	−0.26	−0.21	−0.27
Gender differences in divorce duration	−0.51	−0.38	−0.38	−0.31	−0.27	−0.23	−0.17	−0.21
**Widowhood**								
Effect of gender differences in mortality	1.10	1.11	1.18	0.98	0.93	0.99	1.12	1.13
Effect of gender difference in the proportion of the widowed	5.89	5.55	5.30	4.91	4.67	4.45	4.55	4.17
Gender differences in widowhood duration	6.99	6.67	6.49	5.90	5.60	5.44	5.67	5.30

### Trends and gender differences in marriage duration

**Figure 2:**
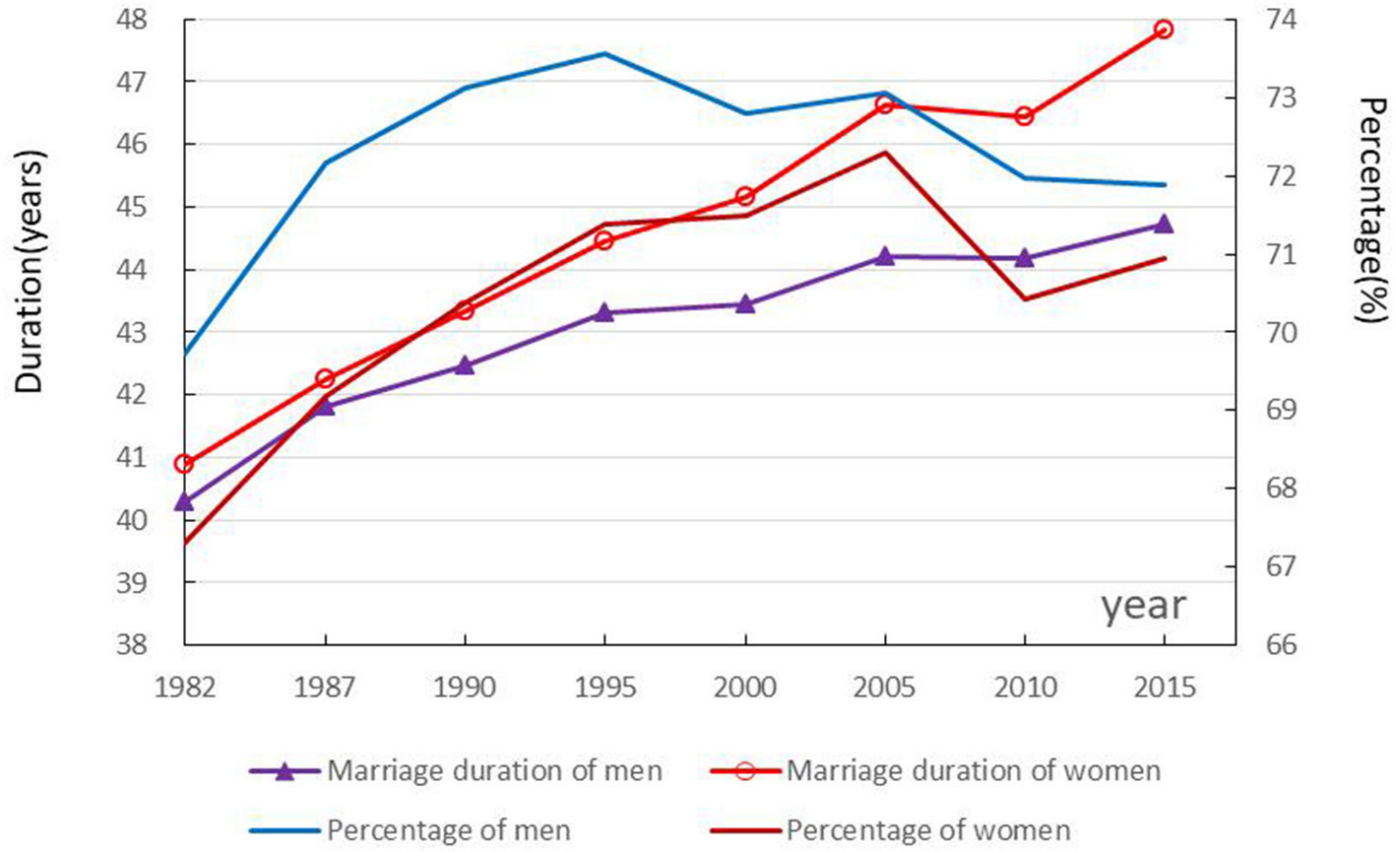
Trends in marriage duration and proportion among 15-year-olds.

The marriage duration extended for both men and women in China in the 1982–2015 period, with women longer than men. However, the proportion of marriage duration in life expectancy at age 15 increases and then decreases, with men larger than women. As shown in Figure 2, the marriage duration of 15-year-old men and women gradually extended from 40.28 and 40.89 years in 1982 to 44.73 and 47.84 years in 2015, respectively. The marriage duration proportion of 15-year-old men increased from 69.73% in 1982 to 73.57% in 1995, and then decreased to 71.88% in 2015, while that of women increased from 67.30% in 1982 to 72.30% in 2005, then declined with fluctuation to 70.94% in 2015.

Increase in marriage duration between 1982 and 2015 was 4.46 and 6.94 years for men and women aged 15 years, respectively. Decomposed results in Table 2 show that decreasing mortality for men and women aged 15 and older has a large positive effect on the extension of the marriage duration, which was 3.49 and 3.95 years, respectively. A rise in the proportion of married men and women aged 45 and older also have a large positive effect on the extension of marriage duration, which was 2.83 and 6.11 years, respectively. However, a drop in the proportion of married men and women aged 15–44 have a large negative effect on the extension of the marriage duration, which was 1.86 and 3.13 years, respectively. The total effects of gender differences in the proportion of married men and women aged 15 and older had a smaller positive effect, with a marriage duration of 0.96 and 2.99 years, respectively.

Gender difference in marriage duration of the 15-year-olds in China fluctuated and increased from 0.61 years in 1982 to 3.10 years in 2015. The decomposition results provided in Table 3 show that the gender difference in mortality had a large positive effect on the gender difference in marriage duration. The effect in mortality increased from 1.80 years in 1982 to 3.94 years in 2015. The gender difference in the married proportion of the population aged 15–49 also had a large positive effect on the gender difference in the marriage duration, but the effect declined, from 3.64 years in 1982 to 2.33 years in 2015. The gender difference in the married proportion of the population aged 50 and older had a large negative effect on the gender difference in marriage duration, and the effect weakened from 4.83 years in 1982 to 3.18 years in 2015. The gender difference in the married proportion of the population aged 15 and above had a negative effect on the gender difference in the married duration, which was relatively stable.

### Trends and gender differences in divorce duration

**Figure 3:**
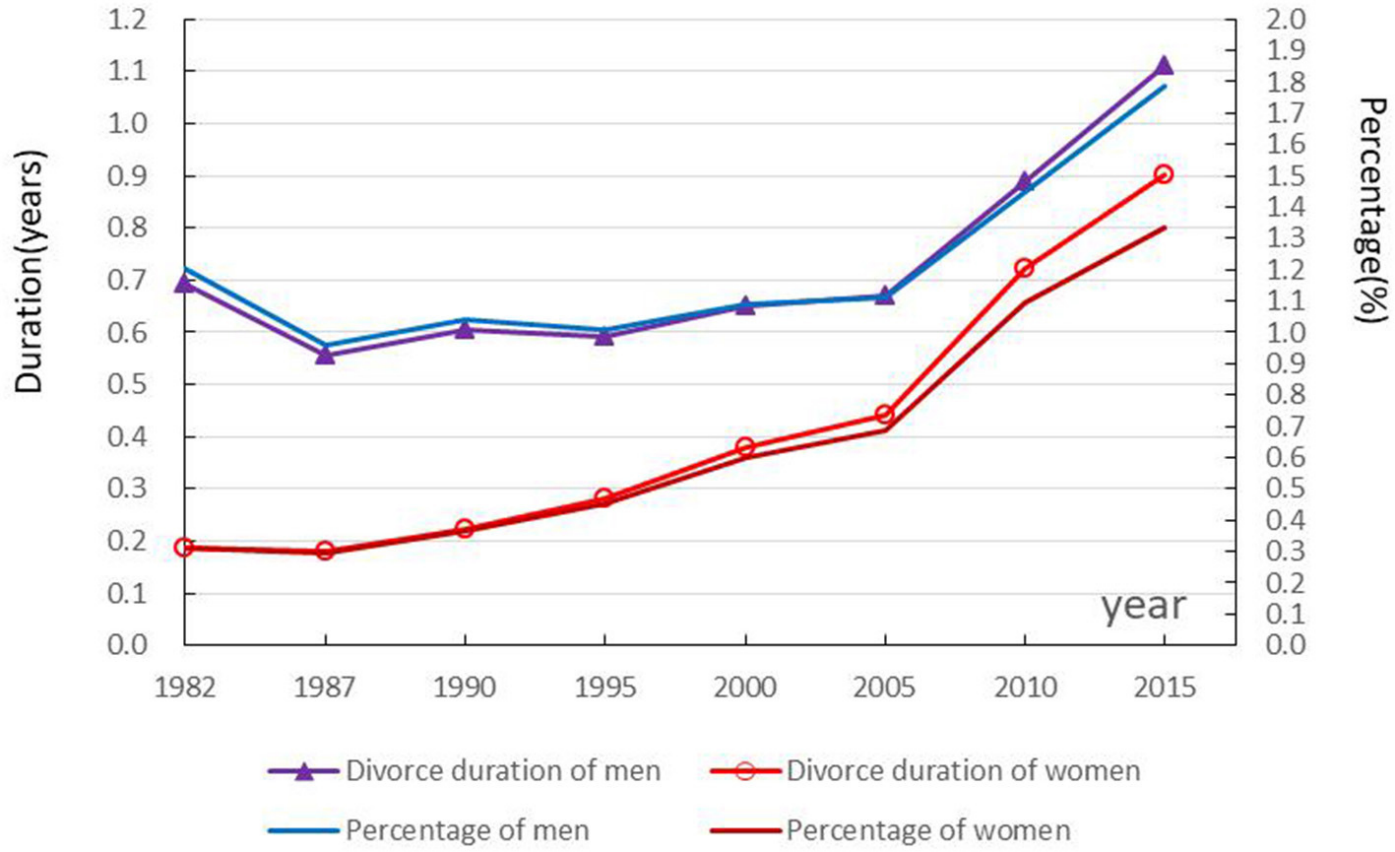
Trends in divorce duration and proportion among 15-year-olds.

The 1982–2015 divorce duration and its proportion in life expectancy for Chinese men and women aged 15 first slightly declined and then rose, but both data of men were higher than those of women. As shown in Figure 3, the divorce duration of 15-year-old men and women decreased from 0.69 years and 0.19 years in 1982 to 0.56 years and 0.18 years in 1987, and then gradually increased to 1.11 years and 0.90 years in 2015, respectively. The proportion of divorce duration of 15-year-old men and women decreased from 1.20 and 0.31% in 1982 to 0.93 and 0.30% in 1987 and then gradually increased to 1.78 and 1.34% in 2015, respectively.

The divorce duration of 15-year-old and above men and women declined by 0.13 and 0.01 years during 1982–1987, but rose by 0.55 and 0.72 years during 1987–2015, respectively. Decomposed results provided in Table 2 show that changes in the proportion of divorce had a large positive effect on those in divorce duration. Decreases in the proportion of divorce men and women contributed to the reduction of 0.18 and 0.01 years of divorce duration from 1982 to 1987. Increases in the proportion of divorce men and women supported the increase of 0.49 and 0.68 years of divorce duration. Declining mortality had a smaller positive effect on longer divorce duration.

The gender difference in divorce duration among 15-year-olds in China shrank from 0.51 years in 1982 to 0.21 years in 2015. Decomposed results in Table 3 show that the gender difference in the proportion of divorce had a large positive effect on that of divorce duration, decreasing from 0.54 years in 1982 to 0.27 years in 2015. However, the gender difference in mortality had a smaller negative effect, leading to a slightly extended divorce duration.

### Trends and gender differences in widowhood duration

**Figure 4:**
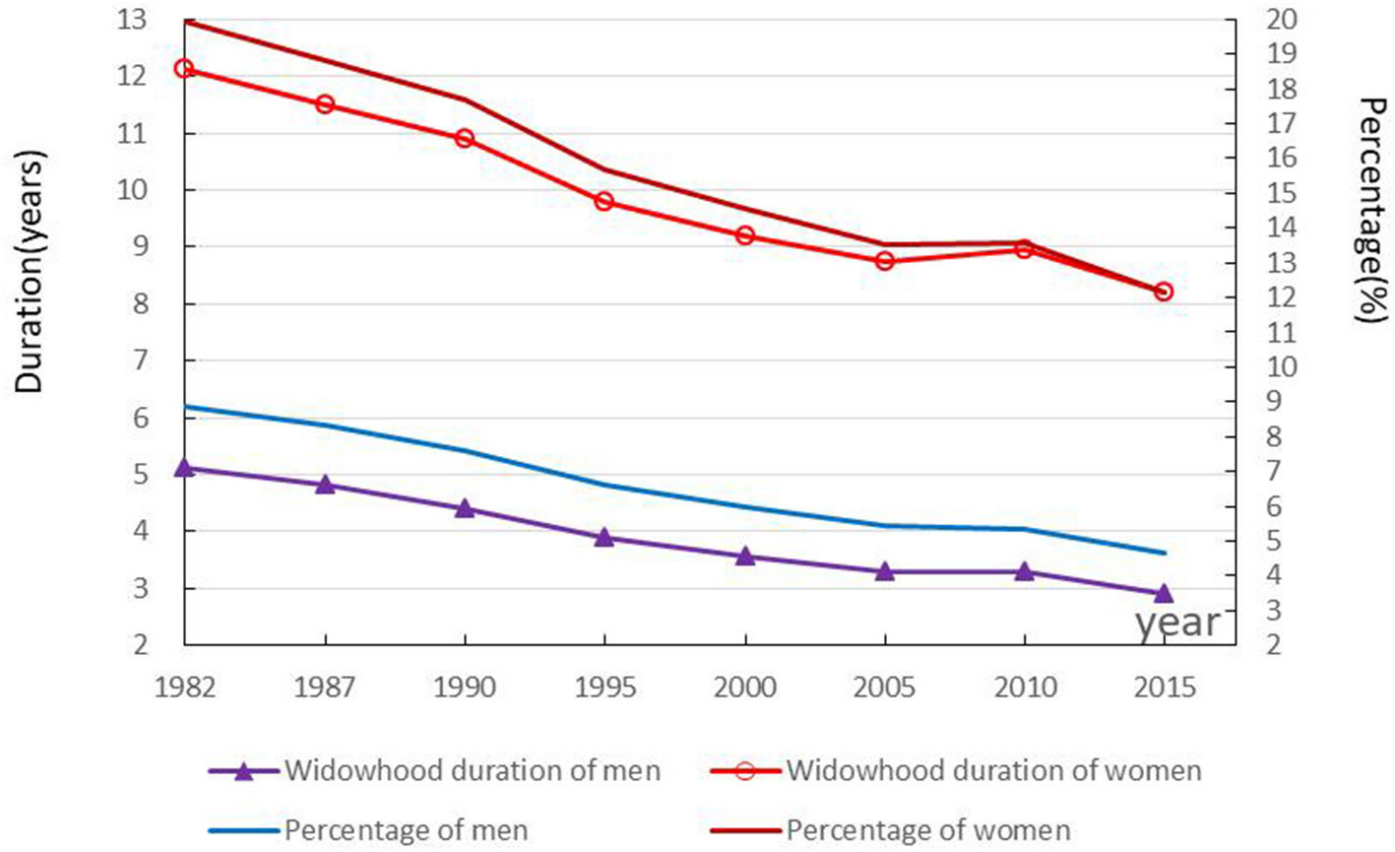
Trends in widowhood duration and proportion among 15-year-olds.

The 1982–2015 widowhood duration and its proportion in life expectancy at age 15 were declining, with both data of women higher than that of men. As shown in Figure 4, the widowhood duration of 15-year-old men and women gradually declined from 5.12 and 12.11 years in 1982 to 2.90 and 8.20 years in 2015, respectively. The widowhood proportion gradually dropped from 8.87 and 19.94% in 1982 to 4.67 and 12.16% in 2015, respectively.

The widowhood duration of 15-year-old men and women contracted by 2.22 and 3.91 years, respectively, from 1982 to 2015. Decomposed results in Table 2 show that the decrease in the proportion of widowed men and women had a large positive effect on the decrease in widowhood duration of 2.96 and 6.56 years, respectively. The results also show that the decrease in mortality had a negative effect on the increase in widowhood duration, which was 0.74 and 2.65 years, respectively.

The gender difference in widowhood duration of the 15-year-old population in China decreased from 6.99 years in 1982 to 5.30 years in 2015. The decomposed results provided in Table 2 show that gender difference in the proportion of the widowed had a significant positive effect on widowhood duration, decreasing from 5.89 years in 1982 to 4.17 years in 2015. The gender difference in mortality has a smaller positive effect, with widowhood duration decreasing from 1.10 years in 1982 to 1.13 years in 2015.

## Discussion

In this study, we first measured the single, marriage, divorce, and widowhood duration of Chinese men and women from 1982 to 2015 with the Sullivan method and analyzed the changing trends and gender differences, and then we decomposed the temporal and gender differences. We drew the following conclusions.

Firstly, the single duration and its proportion in life expectancy of men and women slightly dropped and then raised, with the values of men higher than women, showing a narrowing trend in gender difference. Marriage policy adjustments, socioeconomic development, and increased educational attainment are responsible for the single duration extension. The Marriage Law of the People's Republic of China (1980) stipulated the youngest age of marriage−22 for men and 20 for women—younger than the average age of people's first marriages at that time, reflecting that Chinese people's actual average age of first marriages was older (56, 57). The policy lost efficacy since 1987 at a time when economic and educational development played the primary role in postponing people's age of first marriage. Especially after colleges and universities expanded enrollment in 1999, women's education level increased rapidly, with the proportion of female college students exceeding 50% in 2009 and more in the following years (58). More women chose to postpone their marriage age in a transition faster than that of men. Consequently, the gender difference in single duration narrowed. The single-duration extension further reduced fertility levels and intensified the population's aging (59–61). The rising cost of marriage was one of the main factors prolonging the single duration from 1990 to 2015. Especially in rural areas, most of the cost on wedding house, bride price and marriage expenses are provided by the groom and his parents, while the family has to postpone the son's marriage in order to accumulate these wealth (62, 63).

Secondly, the marriage duration of men and women is extending, with the values of women greater than that of men, demonstrating a widening gender difference gap. However, the proportion of marriage duration in life expectancy rose and then fell, with the values of men higher than that of women. Longer life expectancy is the main factor for the extension of marriage duration. Extension of life expectancy also led to a decrease in the proportion of the widowed and an increase in the proportion of the married annually. Chinese women have a longer marriage duration than men, but men have a higher proportion of marriage duration than women. Two main reasons explain this phenomenon: First, the gender difference in life expectancy is larger than that in marriage duration. For example, the gender difference in life expectancy among 15-year-olds ranged from 3 to 6 years in 1982–2015 (8, 9), while the gender difference in marriage duration ranged from 0.4 to 3.0 years. Second, husbands are on average 2–3 years older than their wives (64). The proportions of marriage duration of both men and women have been declining after 2005, mainly due to people's delayed marriage resulting from higher education levels and the declining proportion of married young people. It is likely that the gender difference in marriage duration will further widen as the marriage squeeze for men continues to intensify (65, 66); the proportion of marriage duration will continue to decrease with more people struggling for higher education levels and thus further postponing marriage.

Thirdly, the divorce duration and its proportion in life expectancy of men and women slightly decreased and then increased, while the data of women were increasing but were still smaller than men's, demonstrating a narrowing gender difference. 1982–1987 saw a decline in men and women's divorce duration due to a decline divorce proportion of men and women. Although divorce rates were rising (67) remarriage rates were also increasing (68), leading to a drop in divorce proportion, from 9.2 and 2.5‰ in 1982 to 7.5 and 2.4‰ in 1987 among men and women aged 15 and older (8, 45). Rapid economic development brought a continuous extension of divorce duration and rise of divorce proportion in 1987–2015. Economic development is the main influencing factor of divorce and is even more influential over time (69, 70). The divorce proportion of men and women aged 15 and older grew from 7.5 and 2.5‰ in 1987 to 19.4 and 14.9‰ in 2015, respectively (9, 45). The main reason divorce duration is longer for men than women is because divorced women can easily remarry compared to men; due to the men's marriage squeeze in China (71), especially in rural areas (72). As women's education level and income improve, highly educated urban women increasingly remain single after divorce (73), resulting in a shorter divorce duration for women than men and a narrowing of the gender difference. The equalization of gender roles and the change in ideology are the main reasons for the increase of divorce duration and the narrowing of gender differences (1). On the one hand, the equalization of gender roles gradually improves women's social and family status, and more and more women take the initiative to divorce. On the other hand, with the change in ideology, people gradually accept the behavior of divorce. More and more couples, even with children involved, are choosing to divorce for their own happiness.

Fourthly, widowhood duration of men and women and its proportion are declining, with the value of women higher than men, leading to narrowing gender difference. Men have a higher mortality rate than women, resulting in a longer duration of widowhood for women. Declining mortality rates led to the shrinkage of widowhood duration. The narrowed gender difference in mortality also shortened widowhood duration. Although data showed the shrinkage of widowhood duration, the size of the widowed elderly is increasing (29, 74). Economic stress, cognitive function, mental health (75–77) and other issues of the widowed elderly may occur and requires more attention.

There are several limitations in this paper. This first is data quality. Underreporting of deaths exist in the 1982–2015 mortality data, and no age-specific marriage data of the elderly aged 60 and above in most years are found, which may affect the results, but this does not affect the judgment of trends and gender differences. The second is the method. A multiple increment-decremental life table method is an ideal tool for this analysis as in previous research. However, it is difficult to obtain the rate of first marriage, divorce, widowhood, remarriage after divorce, remarriage after widowhood, and mortality of people with different marital statuses at different ages, so we adopted the Sullivan method to study the marital duration problem. The third is that cohabitation was not included in the analysis scope. Cohabitation is a significant feature of the second demographic transition in the world (78), which has also emerged in China (79). But as it was impossible to obtain related data to be combined with the census data, we did not consider cohabitation status.

## Data availability statement

The original contributions presented in the study are included in the article/supplementary material, further inquiries can be directed to the corresponding author/s.

## Author contributions

YZ and QJ designed the study and drafted the first version. XY revised the manuscript. All authors contributed to the article and approved the submitted version.

## Funding

This work was jointly supported by the Program of Philosophy and Social Science Foundation of Guangdong in China (GD21CSH06), Youth Project of Ministry of Education Humanities and Social Sciences Fund in China (21YJC840021), and Project of National Social Science Foundation of China (19BRK028).

## Conflict of interest

The authors declare that the research was conducted in the absence of any commercial or financial relationships that could be construed as a potential conflict of interest.

## Publisher's note

All claims expressed in this article are solely those of the authors and do not necessarily represent those of their affiliated organizations, or those of the publisher, the editors and the reviewers. Any product that may be evaluated in this article, or claim that may be made by its manufacturer, is not guaranteed or endorsed by the publisher.
